# Cardiac Rehabilitation in Severe Heart Failure Patients with Impella 5.0 Support via the Subclavian Artery Approach Prior to Left Ventricular Assist Device Implantation

**DOI:** 10.3390/jpm13040630

**Published:** 2023-04-03

**Authors:** Miho Shimizu, Hiroaki Hiraiwa, Shinya Tanaka, Yohei Tsuchikawa, Ryota Ito, Shingo Kazama, Yuki Kimura, Takashi Araki, Takashi Mizutani, Hideo Oishi, Tasuku Kuwayama, Toru Kondo, Ryota Morimoto, Takahiro Okumura, Hideki Ito, Tomo Yoshizumi, Masato Mutsuga, Akihiko Usui, Toyoaki Murohara

**Affiliations:** 1 Department of Rehabilitation, Nagoya University Hospital, Nagoya 466-8560, Japan; 2 Department of Rehabilitation, Mie University Hospital, Tsu 514-8507, Japan; 3 Department of Cardiology, Nagoya University Graduate School of Medicine, Nagoya 466-8550, Japan; 4 Department of Cardiac Surgery, Nagoya University Graduate School of Medicine, Nagoya 466-8550, Japan

**Keywords:** cardiac rehabilitation, severe heart failure, mechanical circulatory support, Impella, left ventricular assist device, subclavian artery, grip strength, knee extension isometric strength

## Abstract

Impella 5.0 circulatory support via subclavian artery (SA) access may be a safe approach for patients undergoing cardiac rehabilitation (CR). In this case series, we retrospectively analyzed the demographic characteristics, physical function, and CR data of six patients who underwent Impella 5.0 implantation via the SA prior to left ventricular assist device (LVAD) implantation between October 2013 and June 2021. The median age was 48 years, and one patient was female. Grip strength was maintained or increased in all patients before LVAD implantation (pre-LVAD) compared to after Impella 5.0 implantation. The pre-LVAD knee extension isometric strength (KEIS) was less than 0.46 kgf/kg in two patients and more than 0.46 kgf/kg in three patients (unavailable KEIS data, *n* = 1). With Impella 5.0 implantation, two patients could ambulate, one could stand, two could sit on the edge of the bed, and one remained in bed. One patient lost consciousness during CR due to decreased Impella flow. There were no other serious adverse events. Impella 5.0 implantation via the SA allows mobilization, including ambulation, prior to LVAD implantation, and CR can be performed relatively safely.

## 1. Introduction

Despite heart transplantation being the preferred treatment option for patients with advanced heart failure (HF), donor shortages limit the use of this strategy [1]. Implantable left ventricular assist devices (LVADs) are an important alternative to managing such patients, and Japanese registry data show that LVAD implantation is employed in approximately 80% of patients who are on the waiting list for heart transplantation [1].

In patients who have undergone LVAD implantation, cardiac rehabilitation (CR) in the acute and chronic postoperative phases improves physical function and independent daily living [2,3]. A recent meta-analysis demonstrated that exercise-based CR improves functional capacity in patients with HF who have undergone LVAD implantation [4]. Another recent study [5] examined the benefits of CR in patients with HF with LVADs versus those of patients who underwent heart transplantation. The study demonstrated similar benefits between the two groups of patients. Specifically, a 6 min walking distance (6-MWD) resulted in the same functional recovery in the two groups, suggesting that CR programs improve the functional capacity of HF patients, regardless of whether or not the patients have undergone heart transplantation or LVAD implantation. 

Other studies have shown that indices related to patients’ preimplantation muscle strength and skeletal muscle mass are associated with postoperative recovery and long-term prognosis [6,7,8,9]. Therefore, CR prior to LVAD implantation is considered important. Approximately 50% of patients prior to LVAD implantation require mechanical circulatory support (MCS) [1,10]. Of the methods used for MCS, intra-aortic balloon pumping (IABP) and veno–arterial extracorporeal membrane oxygenation (ECMO) are usually performed via the femoral artery approach from the groin, which requires bed rest. Moreover, when MCS is prolonged due to severe HF, significant muscle weakness can occur. In addition to muscle weakness, bed rest has systemic disadvantages, such as decreased orthostatic tolerance, increased respiratory complications, and decreased psychocognitive function [11]. 

The Impella device has been available in Japan since 2017 [12], and it has shown promise in circumventing the issues associated with the femoral artery-based, invasive approach to short-term circulatory support. Impella is essentially a short-term circulatory support device [13] that can be implanted via the subclavian artery (SA), which permits rehabilitation, such as standing and walking. Implantation via the SA approach overcomes some of the issues associated with femoral artery implantation, such as lower limb ischemia and the need for bed rest [14,15]. 

Considering the above evidence, it is conceivable that circulatory assistance using the Impella device via the SA approach in patients requiring preoperative adjunctive circulatory management before LVAD implantation may be useful in maintaining or improving physical function. However, to the best of our knowledge, there is a paucity of data on this topic. The purpose of this case series was to retrospectively analyze the data of patients with HF who underwent Impella 5.0 implantation via the SA approach with CR prior to LVAD implantation and to assess their physical function.

## 2. Materials and Methods

### 2.1. Study Population

This was a single-center, observational, retrospective cohort study. The eligibility criteria were consecutive patients who underwent LVAD implantation (HeartMate II^®^ or HeartMate 3^®^, Abbott, Chicago, IL, USA) as a bridge to heart transplantation at Nagoya University Hospital between October 2013 and June 2021. The selection criteria were patients who underwent Impella 5.0 implantation via the SA before LVAD implantation. The decision to place the Impella device was based on the degree of cardiogenic shock and the severity of left-sided HF. Specifically, Impella catheterization was used for left-sided HF that was not adequately supported by IABP or VA-ECMO. The study protocol complied with the Declaration of Helsinki and was approved by the ethics review board of Nagoya University Hospital (approval number: 2021-0397). The requirement for written informed consent was waived due to the retrospective nature of the study, and the patients were given the option to opt out via the Nagoya University Hospital website.

### 2.2. Primary Outcomes

The primary outcomes were grip strength and knee extensor isometric strength (KEIS). The data were collected immediately after Impella 5.0 implantation (post-Impella 5.0), immediately before LVAD implantation (pre-LVAD), immediately after LVAD implantation (post-LVAD), and at hospital discharge. Grip strength and KEIS measurements were not available when the patient’s level of consciousness was decreased or the circulatory status was unstable. Moreover, KEIS was difficult to measure when the patient was unable to sit on the edge of the bed. 

Grip strength was measured in the second position using the JAMAR digital grip strength meter (Patterson Medical, Warrenville, IL, USA). The position for grip strength measurement was supine or sitting, with 90° elbow flexion and a mid-wrist joint position. Grip strength was measured twice on each side, and the maximum value was used [16]. Grip strength (% body weight) was calculated as the ratio of grip strength to body weight.

KEIS was measured using an isometric muscle strength meter (µTas F-1; Anima, Tokyo, Japan). The patients were placed in an end-sitting position on the bed with the hip and knee joints flexed at an angle of 90°. The isometric electromyography electrode was set on the anterior surface of the distal lower leg and fixed to the bar of the bed. The patients performed the exercise once, and the maximum KEIS was measured twice for 5 s each time on the right and left sides. KEIS was analyzed by dividing the maximum value of the two measurements on each side by the body weight on the same day as the measurements were taken [17]. KEIS has a cut-off value of 0.46 kgf/kg to predict exercise capacity at 5 metabolic equivalents in patients with coronary artery disease [18].

### 2.3. Clinical Parameters

Demographic information, echocardiographic findings prior to Impella 5.0 implantation, preoperative assisted circulation, surgical information, the postoperative course, and CR information were obtained from patients’ medical records. The length of MCS prior to Impella 5.0 implantation was calculated including the period when MCS was required at the hospital prior to admission to Nagoya University Hospital. Regarding postoperative information, the post-LVAD ventilator management time referred to the diagnostic criteria of the Japan Cardiovascular Surgery Database, and when intubation and extubation were repeated, the ventilator management time was totaled [19]. Among the CR information, CR with Impella 5.0 was assessed using the intensive care unit mobility scale (IMS) [20]. To assess the level of mobility 1 week prior to LVAD implantation, the maximum amount of time (in minutes per day) in IMS 3 (sitting on the edge of the bed), as well as neuromuscular electrical stimulation (NMES), was recorded. The post-LVAD CR data were collected, including the presence or absence of NMES, days to sitting, days to 100 m gait, and days to the exercise room. 

### 2.4. CR with Impella 5.0 Support

The preoperative and postoperative CR programs were based on the guidelines for the treatment of acute and chronic heart failure [21] and the guidelines for the treatment of implantable assistive devices for severe heart failure [22].

CR before Impella 5.0 implantation and post-Impella 5.0 was performed with passive and active exercise and NMES considering the general condition of the patients. CR was performed 5–6 days per week. NMES was performed using SOL-1 (Minato Medical Science, Osaka, Japan). For patients who were conscious, active exercises were performed, but for patients who were sedated or not fully conscious, CR was performed with passive exercises. Previous studies have shown that NMES is safe in patients with acute HF [23] and that it can be performed safely with Impella and LVAD implantation [24,25]. It is recommended that NMES be considered for patients who have difficulty performing exercise therapy [26]. The protocol used at our hospital for CR during Impella support is shown in Table S1. When the Impella 5.0 device was inserted via the SA, out-of-bed mobilization was considered, and CR was performed in collaboration with multiple professionals, including physicians, nurses, clinical engineers, and physical therapists. Each patient received sitting on the edge of the bed, standing, wheelchair sitting, walking, seated or standing lower extremity muscle strengthening exercises, and a seated ergometer, depending on general stability and muscle strength and endurance. For rehabilitation on the bed, the cardiologist or clinical engineer was not at the bedside; the physical therapist and nurse were at the bedside. Away from the bed (IMS ≥ 3), cardiologists, clinical engineers, physical therapists, and nurses were at the bedside during rehabilitation. The most important points of CR with Impella 5.0 support are the Impella position check by the physician before and after mobilization and the Impella waveform and alarm check by the clinical engineer.

### 2.5. Statistics

The data are presented by case, and some continuous data are expressed as median (interquartile range (IQR)). All statistical analyses were performed using SPSS statistical software, version 28.0 (IBM Corp., Armonk, NY, USA).

## 3. Results

### 3.1. Patients’ Baseline Characteristics

A total of 50 consecutive patients with severe HF required LVAD implantation as a bridge to heart transplantation. Of these, six patients underwent Impella 5.0 implantation via the SA approach. The characteristics of these patients are shown in Table 1. The median age of the patients was 48 years (IQR: 39–58 years), and there was one female patient. The etiologies of HF were as follows: dilated cardiomyopathy (*n* = 2), ischemic cardiomyopathy (*n* = 2), cardiac sarcoidosis (*n* = 1), and triglyceride deposit cardiomyovasculopathy (*n* = 1). At the time of Impella 5.0 implantation, all patients were using transvenous inotropic agents. The duration of MCS prior to Impella 5.0 implantation was 3–27 days.

### 3.2. Post-Impella 5.0 and Pre-/Post-LVAD Surgery

The implantation period for Impella 5.0 ranged from 10 to 97 days (Table 2). The pre-LVAD hospital stay duration ranged from 43 to 129 days. Pre-LVAD adverse events included the need for Impella replacement (nos. 1, 2, and 4), hemorrhage (nos. 4 and 6), and loss of consciousness due to decreased Impella flow during CR (no. 4). Post-LVAD, the median intensive care unit stay was 21 days (IQR: 10–37 days), and the median hospital stay was 159 days (IQR: 79–202 days]).

### 3.3. CR with Impella 5.0 Support

**Figure 1:**
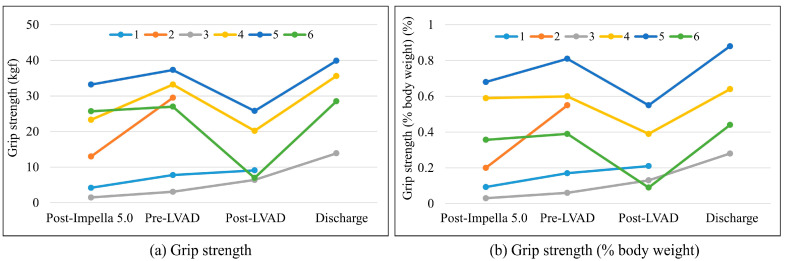
Change in (**a**) grip strength and (**b**) grip strength (% body weight).

The preoperative and postoperative CR data are presented in Table 3. Grip strength was maintained or improved from post-Impella 5.0 to pre-LVAD in all patients. Although grip strength declined post-LVAD in three patients, it was improved at hospital discharge (Figure 1). The median grip strength (% body weight) was 27.7% post-Impella 5.0, 47.1% pre-LVAD, 21.3% post-LVAD, and 53.7% at hospital discharge (Figure 1). Of the five patients for whom KEIS could be measured pre-LVAD, two had a KEIS of less than 0.46 kgf/kg and three had a KEIS of more than 0.46 kgf/kg. Of these five patients, three demonstrated a maintenance of or improvement in KEIS post-LVAD or at discharge. The three patients who were discharged and sent home had a 6-MWD of 370–540 m. NMES was performed in the two patients with a low post-Impella 5.0 and pre-LVAD grip strength and KEIS. In the week prior to LVAD implantation, five patients were classed as having IMS ≥ 3 (83%), and two patients were classed as ambulatory IMS 9 (33%). The walking distance of the two patients classed as IMS 9 ranged from 80 to 120 m. Of the five patients who were able to get out of bed post-LVAD, four patients could sit on the edge of the bed at 3–7 days, and three patients were able to walk 100 m. One patient with low pre-LVAD physical function took 54 days after surgery to walk 100 m, while the other two patients could walk 100 m at 11–12 days after surgery.

Adverse events during CR included loss of consciousness in one patient. While sitting in a wheelchair and taking physical measurements after removing the elastic bandage attached to the lower leg, flow decreased and the waveform of Impella flattened, resulting in hypotension and loss of consciousness. When the patient was placed in bed, they quickly regained consciousness, and there was no Impella malposition. No other adverse events were observed during CR, and the patient was able to practice walking.

**Table 1:** Patient demographics, clinical characteristics, and interventions.

No.	Age	Sex	BMI	HF Etiology	LVEF, %	Comorbidities	Oral Medications Prior to Impella 5.0 Implantation	MCS Prior to Impella 5.0 Implantation	Length of MCS Prior to Impella 5.0 Implantation, Days
1	37	M	20.9	TGCV	10.9	CKD, myopathy	ARB, BB, MRA, loop diuretic	IABP, VA-ECMO	27
2	42	M	25.1	ICM	16.6	PAD, dyslipidemia	Antiplatelet agent, statin	IABP, VA-ECMO	11
3	58	F	22.7	ICM	20.0	Diabetes mellitus, dyslipidemia, post-CABG, post-SVR	Antiplatelet agent, statin, BB, MRA, loop diuretic	IABP	23
4	38	M	22.5	DCM	14.0	Bronchial asthma, chronic hepatitis	ACEi, BB, loop diuretic	IABP	8
5	55	M	18.2	DCM	16.0	None	BB, loop diuretic	Impella CP, VA-ECMO	5
6	54	M	23.8	Cardiac sarcoidosis	20.5	Hypertension	None	Impella 2.5, VA-ECMO	3

ACEi, angiotensin-converting enzyme inhibitor; ARB, angiotensin II receptor blocker; BB, beta-blocker; BMI, body mass index; CABG, coronary artery bypass graft; CKD, chronic kidney disease; DCM, dilated cardiomyopathy; HF, heart failure; IABP, intra-aortic balloon pumping; ICM, ischemic cardiomyopathy; LVEF, left ventricular ejection fraction; MCS, mechanical circulatory support; MRA, mineralocorticoid receptor antagonist; PAD, peripheral arterial disease; SVR, surgical ventricular restoration; TGCV, triglyceride deposit cardiomyovasculopathy; VA-ECMO, veno–arterial extracorporeal membrane oxygenation.

**Table 2:** Details of the treatment course, adverse events, and reasons for discharge.

No.	Length of Impella 5.0, Days	Pre-LVAD Mechanical Ventilation, Days	Pre-LVAD Hospital Stay, Days	LVAD Operative Time, Hours	Post-LVAD ICU Stay, Days	Post-LVAD Adverse Events	Discharge
1	91	93	129	9.2	203	Exchange of LVAD, wound infection, stroke	Hospital death
2	97	11	99	6.8	32	Pump thrombosis and hypoxic–ischemic encephalopathy	Hospital death
3	39	19	47	6.7	10	Gastrointestinal hemorrhage	Home
4	58	3	65	4.8	10	-	Home
5	43	4	43	5.1	10	Rethoracotomy	Home
6	10	5	69	5.3	39	Stroke	Another hospital

ICU, intensive care unit; LVAD, left ventricular assist device.

**Table 3:** Indices of cardiac rehabilitation post-Impella 5.0 implantation, pre-/post-LVAD implantation, and at discharge.

No.	Pre-LVAD IMS	Grip Strength, kgf				Grip (% Body Weight), %				KEIS, kgf/kg			
		Post-Impella 5.0	Pre-LVAD	Post-LVAD	Discharge	Post-Impella 5.0	Pre-LVAD	Post-LVAD	Discharge	Post-Impella 5.0	Pre-LVAD	Post-LVAD	Discharge
1	1	4.2	7.8	9.1	-	0.09	0.17	0.21	-	-	0.11	-	-
2	9	13.0	29.5	-	-	0.20	0.55	-	-	-	0.47	-	-
3	3	1.5	3.1	6.4	13.9	0.03	0.06	0.13	0.28	-	0.07	-	0.48
4	9	23.3	33.2	20.2	35.6	0.59	0.60	0.39	0.64	-	0.58	0.60	0.58
5	6	33.2	37.3	25.8	39.9	0.68	0.81	0.55	0.88	-	0.50	0.54	0.80
6	3	25.7	27.0	7.0	28.5	0.36	0.39	0.09	0.44	-	-	-	0.42
**No.**	**Six-Minute Walking Distance at Discharge, m**	**NMES**						**Progress of Cardiac Rehabilitation**					
		**Pre-LVAD Total Number of Times**		**Pre-LVAD Intensity of NMES, mA**		**Pre-LVAD Total Number of Times**		**Post-LVAD Sitting, Days**		**Post-LVAD 100-m Gait, Days**		**Post-LVAD Exercise Room, Days**	
1	-	53		65		Yes		21		-		-	
2	-	-		-		No		-		-		-	
3	440	24		40		Yes		3		54		17	
4	540	-		-		No		3		11		19	
5	370	-		-		No		4		12		19	
6	-	-		-		No		7		-		91	

IMS, intensive care unit mobility scale; KEIS, knee extension isometric strength; LVAD, left ventricular assist device; NMES, neuromuscular electrical stimulation.

## 4. Discussion

The present study describes CR and physical function in several patients who underwent Impella 5.0 implantation via the SA with subsequent conversion to LVAD implantation, and presents a protocol for CR with Impella 5.0 support. All but one of the six patients who underwent Impella 5.0 implantation via the SA could sit on the edge of the bed, stand, or ambulate before LVAD implantation, suggesting that CR may be a relatively safe approach that can be performed prior to LVAD implantation.

The features of Impella include effective left ventricular support and pressure load reduction, as well as increased organ perfusion and ease of extubation and ambulation [27]. In addition, Impella facilitates smoother management than extracorporeal LVAD implantation and reduces surgical invasiveness during durable LVAD implantation [28]. In this study, five patients required long-term MCS, such as Impella, IABP, or VA-ECMO, pre-LVAD for a total duration of 43–118 days. CR in patients with Impella implantation via the SA has not been adequately reported. Bed rest is mandatory when MCS via the groin (e.g., the femoral artery approach) is required [29]. Disadvantages associated with bed rest include skeletal muscle atrophy, respiratory complications, and mental deterioration. Regarding the level of exercise in patients with Impella support, a retrospective study in patients with cardiogenic shock showed that the surviving group could stand or walk around 10 steps on average [15]. Impella implantation via the SA approach overcomes some of the issues associated with implantation via the femoral artery approach as it does not require bed rest and eliminates the risk of lower limb ischemia [29]. In this study, assisted circulation with Impella support via the SA allowed many of the patients to be gradually accustomed to the treatment by rehabilitation prior to LVAD implantation, with two reaching IMS 9 ambulation, one reaching IMS 6 standing, and two reaching IMS 3 sitting. Therefore, CR with Impella 5.0 implantation via the SA as opposed to via the femoral artery can be performed relatively safely and allows patients with HF to perform exercise prior to LVAD implantation.

To safely perform CR with Impella support, risk management was performed before, during, and after the intervention through collaboration and role-sharing among multiple disciplines. There was only one adverse event during CR, which was a decrease in Impella flow, leading to loss of consciousness. Possible causes included withdrawal of inotropic agents and a shift in circulating blood volume due to the removal of the elastic bandage. No serious incidents, such as Impella malposition or massive bleeding, occurred during CR [14]. When performing CR with Impella support, it is essential to understand the characteristics of the Impella device and to share goals among multiple disciplines.

Changes in physical function between post-Impella 5.0 and pre-LVAD were difficult to assess due to the small number of patients. However, the median grip strength (%body weight) pre-LVAD was 47.1%, which far exceeds the grip strength of 25% proposed as a criterion for frailty in patients with LVAD implantation [30]. In three of the patients, KEIS pre-LVAD was above 0.46 kgf/kg, which predicts five metabolic equivalents [18]. Several studies have shown that indices related to patients’ muscle strength and skeletal muscle mass before LVAD implantation are associated with postoperative recovery and long-term prognosis [6,7,8,9]. Therefore, CR, including mobilization, with Impella 5.0 support may be useful to prevent the decline in physical function in HF patients before LVAD implantation. A previous case report in an Impella-supported patient reported improved limb strength with NMES and mobilization [24], which supports the results of this study. However, the study differed from the present study in that the patient was able to wean off of Impella and did not require LVAD implantation [24]. Therefore, this study is influential in that it demonstrates the physical functioning of HF patients who undergo Impella 5.0 support with CR before LVAD implantation.

This study has some limitations that should be noted. This study was a retrospective case series, so no conclusions could be drawn regarding the effectiveness of CR. In addition, there were missing data on the assessment of physical function. Moreover, because of the highly individualized nature of HF control during Impella implantation, it was difficult to perform a definitive assessment. However, given the results of the cases that were evaluable and the fact that Impella implantation via the SA allowed mobilization, we suggest that CR can be performed relatively safely in these patients.

## 5. Conclusions

In patients with HF who undergo Impella 5.0 implantation via the SA, CR may be a relatively safe approach that can be performed prior to LVAD implantation, allowing ambulation and other forms of mobilization.

## Data Availability

The deidentified participant data will be shared on a request basis. Please directly contact the corresponding author to request data sharing.

## Supplementary Materials

The following supporting information can be downloaded at: https://www.mdpi.com/article/10.3390/jpm13040630/s1, Table S1: Cardiac rehabilitation with Impella support.
