# Supplementary material for: Cardiac Rehabilitation in Severe Heart Failure Patients with Impella 5.0 Support via the Subclavian Artery Approach Prior to Left Ventricular Assist Device Implantation
Source: J Pers Med. 2023 Apr 3;13(4):630. doi: 10.3390/jpm13040630 (PMC10143331; doi:10.3390/jpm13040630)
Supplement: Supplementary file 1 [file jpm-13-00630-s001.zip › jpm-2291158-supplementary.pdf]

Table S1. Cardiac rehabilitation with Impella 5.0 support

Goals of Rehabilitation

- ・Rehabilitation strategy for treatment after withdrawal from Impella support
- ・Maintenance and improvement in physical function with Impella support
- ・Improvement and prevention of progression of complications that occurred before the start of Impella support
- ・Prevention of new complications, such as infection, atelectasis, and delirium

Protocols for Rehabilitation

Confirmation (shared by multiple professions, explanation and consent to patients, understanding of the conditions and settings of the Impella device)

Medical staff	・Multidisciplinary conferences are held for a team consisting of cardiologists, nurses, clinical engineers, physical therapists, nutritionists, and clinical psychologists<br>・The condition of the patient (whether the condition is rehabilitative, whether the patient is ready to be weaned, and the nutritional and mental status) is assessed<br>・Multidisciplinary staff acknowledge that the patient is eligible for physical therapy
Patients	・The purpose of physical therapy, the individual patient's goals, and the program content are explained to the patient or their family members before obtaining consent for rehabilitation
Impella	・Impella model type and settings<br>・Impella stability (pump flow, purge fluid flow, pump power setting changes)<br>・Impella insertion site (femoral, subclavian, axillary, or other artery)<br>・Impella catheter fixation<br>・Impella-related complications, such as bleeding and hemolysis

Implementation (before, during, and after rehabilitation)

Before	Medical staff	・Confirm the role of each occupation before rehabilitation begins<br>・Cardiologist: ascertain hemodynamics during rehabilitation, confirm the Impella positioning by echocardiography, and confirm what action should be taken in case of any issues<br>・Nurse: manage intravenous route and oxygen, manage other necessary equipment, apply elastic bandage to the patient's lower leg<br>・Clinical engineer: manage Impella equipment, check Impella waveform and alarms<br>・Physiotherapist: assist with bed release, measure rehabilitation and exercise indices<br>・Nutritionist: assess the patient's nutritional status before rehabilitation<br>・Clinical psychologist: assess the patient's mental state

	Patients	･Measurement and evaluation of blood pressure, heart rate, and other hemodynamic indicators with right heart catheterization before rehabilitation<br>･Checking subjective symptoms and other findings before rehabilitation<br>･Wearing elastic bandage on lower leg (if there is a risk of a decrease in Impella pump flow)
	Impella	･Transthoracic echocardiography to confirm Impella pump position and Impella insertion site
During	Medical staff	･Cardiologist: ascertain hemodynamics during rehabilitation, confirm Impella positioning by echocardiography, and confirm what to do in case of any issues<br>･Nurse: manage intravenous route and oxygen, manage other necessary equipment, assist patients during rehabilitation<br>･Clinical engineer: manage Impella equipment, check Impella waveform and alarms<br>･Physiotherapist: implement NEMS, assist with bed release, measure rehabilitation and exercise indices
	Patients	･Measurement and evaluation of blood pressure, heart rate, and other hemodynamic indicators with right heart catheterization during rehabilitation<br>･Checking subjective symptoms and other findings during rehabilitation<br>･Measurement of grip strength and MRC<br>･Measurement of KEIS (if the patient is able to hold a stable sitting position)
	Impella	･Transthoracic echocardiography to confirm Impella pump position and Impella insertion site<br>･Check frequently when changing rehabilitation level (sitting, standing, walking)
After	Medical staff	･Cardiologist: ascertain hemodynamics after rehabilitation, confirm Impella positioning by echocardiography<br>･Nurse: manage intravenous route and oxygen, assist patients after rehabilitation<br>･Clinical engineer: manage Impella equipment, check Impella waveform and alarms<br>･Physiotherapist: assess the patient's condition after rehabilitation
	Patients	･Measure and evaluate blood pressure, heart rate, and other hemodynamic indicators using right heart catheterization during

		rehabilitation<br>・Check subjective symptoms and other findings during rehabilitation
	Impella	・Transthoracic echocardiography to confirm Impella pump position and Impella insertion site

**Trouble Shooting**
・If the Impella pump malpositions or the suction alarm sounds, search for the cause (evaluate for insufficient preload or Impella pump malposition)<br>・Based on the subjective symptoms, other findings, and Impella waveform, decide whether to continue rehabilitation<br>・When a serious event occurs, such as low Impella pump flow, low purge flow, or pump stoppage, stop rehabilitation immediately and stabilize the patient's hemodynamics

KEIS, knee extension isometric strength; MRC, Medical Research Council; NMES, neuromuscular electrical stimulation.
